# The octopus technique: a technical modification for tension-free closure in abdominoplasty

**DOI:** 10.1080/23320885.2026.2648257

**Published:** 2026-04-13

**Authors:** Adnan G. Gelidan, Hatan Mortada, Hisham Alghamdi, Amr Youssef Arkoubi

**Affiliations:** a Division of Plastic Surgery, Department of Surgery, College of Medicine, King Saud University, Riyadh, Saudi Arabia; b Department of Plastic Surgery & Burn Unit, King Saud Medical City and Division of Plastic Surgery, Department of Surgery, King Saud University Medical City, King Saud University, Riyadh, Saudi Arabia; c Division of Plastic Surgery, Department of Surgery, College of Medicine, Imam Mohammad Ibn Saud Islamic University, Riyadh, Saudi Arabia

**Keywords:** Abdominoplasty, tension-free closure, vertical incisions, lipectomy, surgical technique, wound healing, octopus technique

## Abstract

This paper describes the Octopus Technique, a technical modification for tension-free abdominoplasty closure using short vertical relaxing incisions for multidirectional tension redistribution. In 12 consecutive patients, no wound dehiscence or flap necrosis occurred. Preliminary results suggest it is safe and reproducible.

## Introduction

Abdominoplasty, commonly known as a “tummy tuck,” is one of the most frequently performed aesthetic procedures to improve abdominal contour by removing excess skin and fat. According to the 2024 report of the American Society of Plastic Surgeons, approximately 171,064 abdominoplasties were performed in the United States, making it the third most common cosmetic surgical procedure [1]. The success of abdominoplasty depends on patient selection, surgical design, and meticulous wound closure [2].

A key technical challenge in abdominoplasty is achieving a tension-free closure, which is critical for optimal wound healing and prevention of complications such as wound dehiscence, flap necrosis, and hypertrophic scarring [3,4]. Over the years, several modifications have been introduced to reduce closure tension. Progressive tension sutures were developed to distribute forces evenly and minimize seroma formation [3,5]. Mesh reinforcement techniques provide additional support but require foreign material placement, increasing cost and risk of infection [6]. Lipoabdominoplasty with selective undermining improves flap vascularity [7], yet its limited dissection may restrict tissue advancement and contour refinement.

Despite these refinements, none directly address the localized high-tension zones often encountered at the midline and lateral edges during closure. The Octopus Technique was designed to overcome these challenges through a simple, suture-free, multidirectional tension-release modification. It combines a standard transverse incision with short vertical relaxing incisions along the midline and lateral aspects, allowing controlled redistribution of tension in both vertical and horizontal planes.

This configuration differs conceptually from previous methods by permitting real-time intraoperative adjustment of tension without deep fixation sutures or additional materials. While the concept of relaxing incisions is well established in reconstructive surgery, their specific application in this reproducible configuration for abdominoplasty has not been previously described. By enhancing flap mobility and reducing stress at critical closure points, the Octopus Technique aims to minimize wound-related complications and achieve a smoother, more natural contour. The name “Octopus Technique” reflects the incision pattern’s resemblance to the arms of an octopus, symbolizing its multidirectional tension control and adaptability.

## Technique description

### Preoperative preparation and marking

After preoperative counseling and discussion of potential risks, marking is performed with the patient standing upright. The midline is delineated from the xiphoid process to the upper border of the pubic region.

With the patient seated with hips and knees flexed at 90°, the lateral extent of the abdominal skin fold is identified and marked. This position allows accurate assessment of skin redundancy and helps determine the extent of the transverse incision line.

### Patient selection and indications

Patients were selected based on the following inclusion criteria: moderate-to-severe abdominal skin laxity (Matarasso Classification III–IV), BMI < 30 kg/m^2^, and anticipated high closure tension based on preoperative pinch test and skin redundancy assessment. Exclusion criteria included active smoking within 6 weeks of surgery, BMI ≥ 30 kg/m^2^, uncontrolled diabetes mellitus (HbA1c > 8%), prior abdominoplasty, and planned concurrent bariatric procedures. The technique is particularly advantageous in patients with significant vertical skin excess, post-massive weight loss patients undergoing extended abdominoplasty, and combined contouring procedures where tension-free closure may be challenging to achieve with standard methods alone.

### Patient positioning and incision design

The patient is then placed supine on the operating table. The lower transverse incision is planned approximately 5–6 cm above the vulvar commissure (or superior anterior vaginal margin), extending laterally toward the anterior superior iliac spine (ASIS) on both sides.

Two vertical reference lines, each 7 cm lateral to the midline, are marked to guide intraoperative tension assessment. A 10 cm reference point is identified at the ASIS to define the superior-lateral limit of dissection.

### Dissection technique

Following induction of anesthesia and sterile preparation, an inferior incision is made, and dissection proceeds superiorly in the sub-Scarpa’s plane up to the xiphoid process and costal margins. Once adequate flap mobilization is achieved, the table is flexed to the desired angle for closure.

### Tension release and closure

The midline vertical incision above the umbilicus and the two lateral vertical reference lines are opened sequentially, only to the extent required to relieve tension. This selective release permits controlled redistribution of forces and a balanced, tension-free closure. Closure is performed in a layered fashion after ensuring adequate flap redraping and tension release. The deep fascial layer is approximated first using interrupted absorbable sutures to eliminate dead space and evenly distribute tension. The superficial fascia (Scarpa’s layer) is then secured to the underlying fascia to provide additional support and prevent downward scar migration. Skin closure is completed with fine intradermal sutures, ensuring minimal surface tension. This sequence allows gradual tension redistribution from the skin to deeper supportive layers, maintaining a tension-free and well-vascularized closure. Typically, three short relaxing incisions are used: one midline and one lateral incision on each side, approximately 7 cm from the midline.

After confirming appropriate flap redraping, the umbilicus is transposed through a new opening at its natural anatomic position, typically at the level of the iliac crests along the midline. It is secured with interrupted dermal sutures at the base and fine intradermal sutures at the skin edge to achieve natural contour and minimal scar visibility. Unlike fleur-de-lis abdominoplasty, which requires a full vertical excision and results in a long midline scar, the Octopus Technique relies on several short vertical relaxing incisions opened only as needed for tension release. These incisions are temporary tension-reduction points rather than excisional vectors, allowing preservation of a single low transverse scar.

### Outcome assessment

Postoperative complications were recorded prospectively at scheduled follow-up visits (1 week, 2 weeks, 1 month, 3 months, and 6 months postoperatively) and classified according to the Clavien-Dindo classification system (Grade I–V). Minor complications were defined as Grade I–II (requiring no intervention or pharmacological treatment only), and major complications as Grade III–V (requiring surgical, radiologic, or endoscopic intervention, or resulting in organ dysfunction or death).

### Visual summary

This configuration—comprising one transverse and multiple short vertical relaxing incisions—resembles the arms of an octopus, inspiring the name *Octopus Technique*. It enables gradual, site-specific tension release and helps prevent complications such as wound dehiscence, flap edge necrosis, or widened scars.

**Figure 1.**
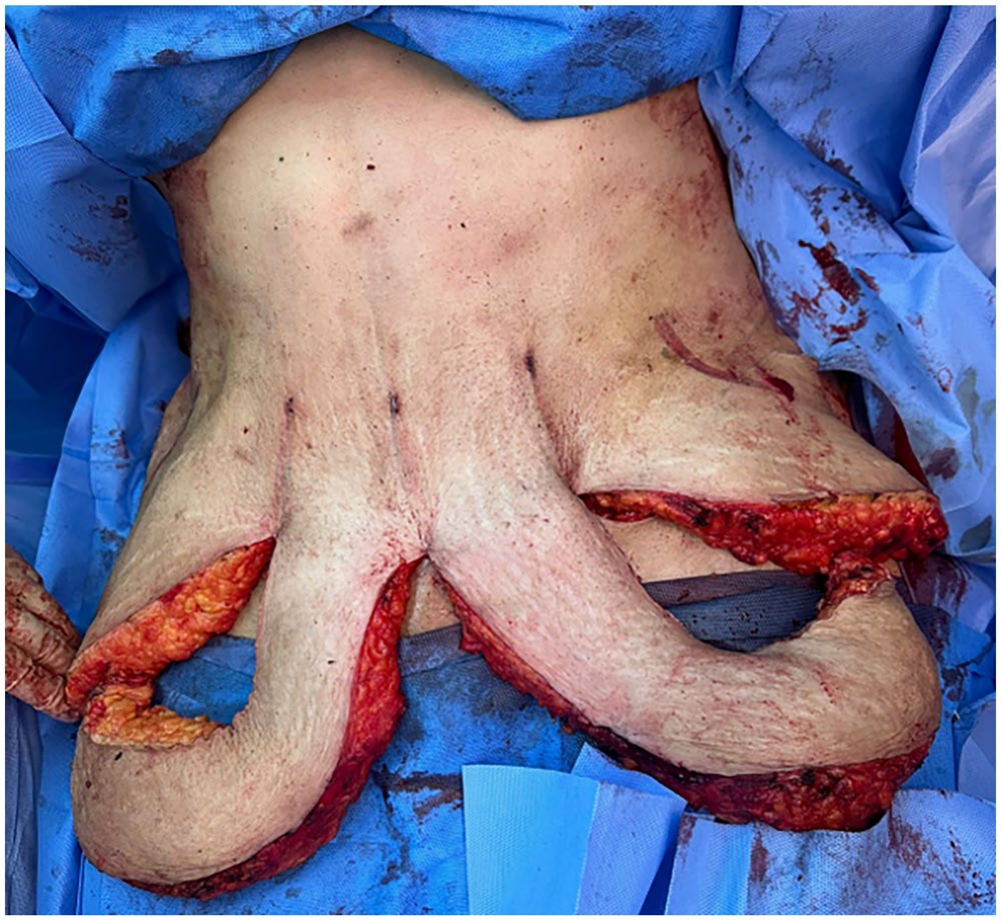
Intraoperative pictures demonstrating the octopus technique for abdominoplasty. The incision pattern, consisting of a transverse incision with additional vertical midline and lateral incisions, resembles the arms of an octopus.

**Figure 2.**
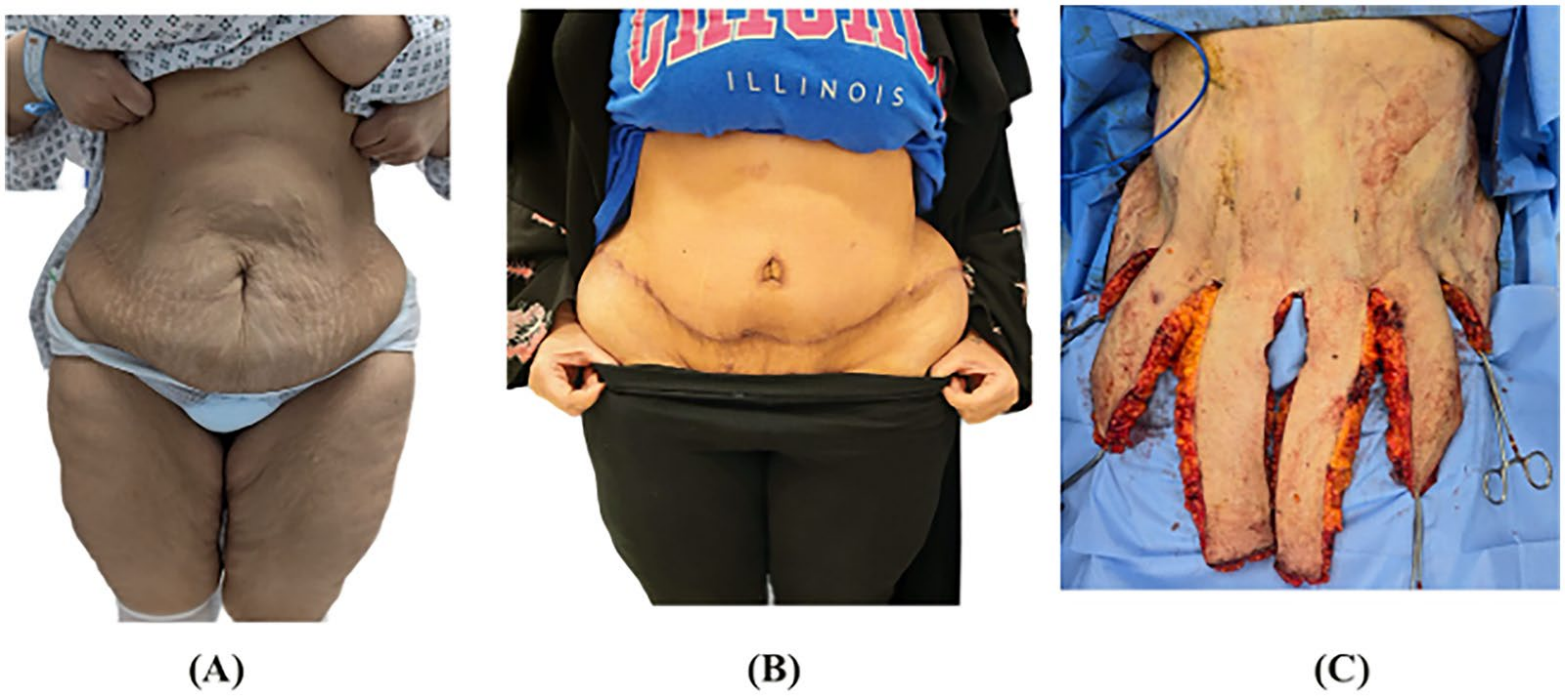
Preoperative (A), postoperative (B), and intraoperative (C) images demonstrating the Octopus Technique. The incision design includes a central vertical incision and multiple lateral vertical extensions branching from the transverse abdominoplasty incision, resembling the arms of an octopus. This configuration enables precise tension distribution and facilitates a meticulous, tension-free closure.

**Figure 3.**
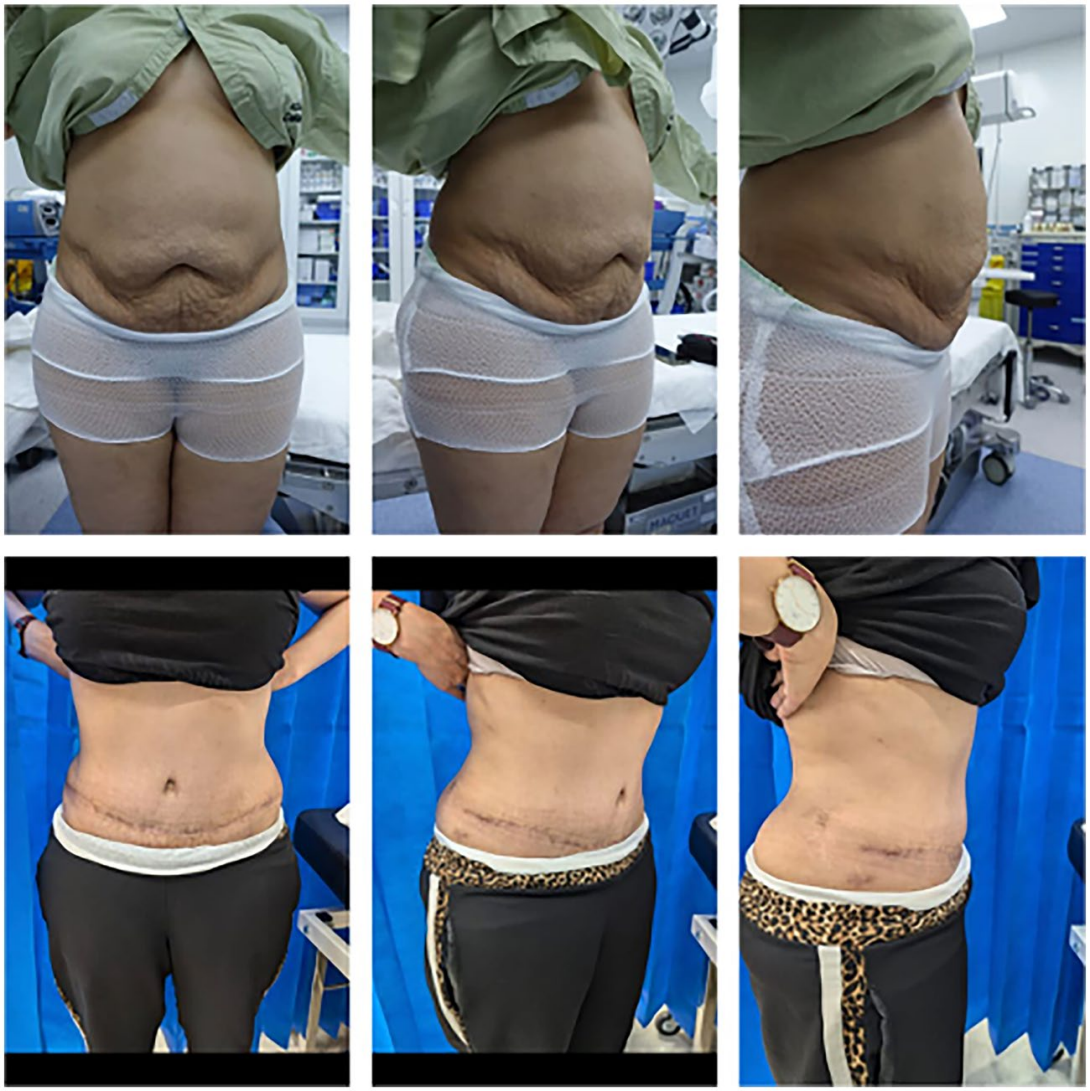
Preoperative (top row) and postoperative (bottom row) clinical photographs of a patient who underwent abdominoplasty using the Octopus Technique. The postoperative images, taken at 3 months, demonstrate a smooth abdominal contour, symmetric scar positioning, and a well-healed tension-free incision with no evidence of wound dehiscence or flap compromise.

**Figure 4.**
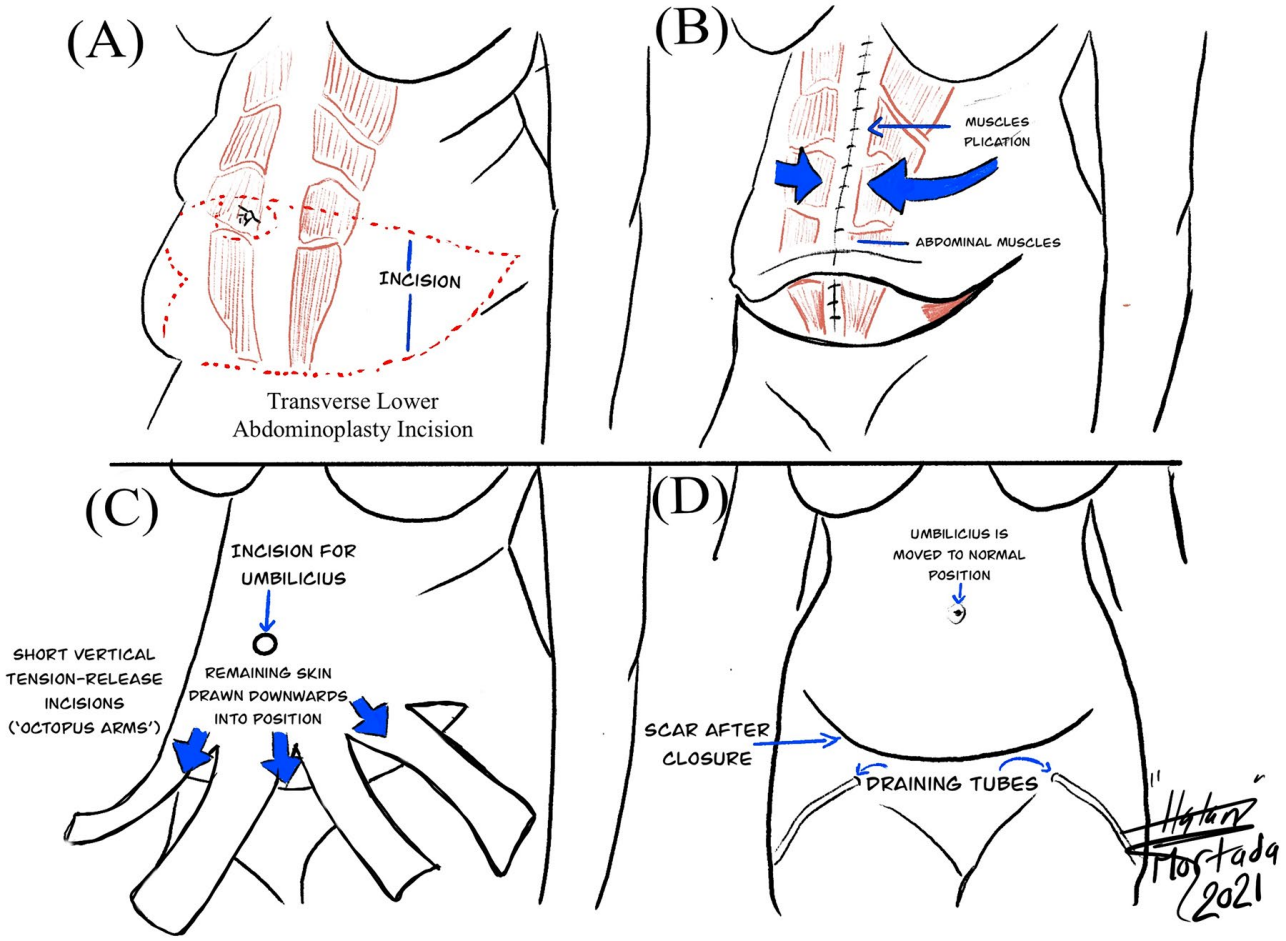
Schematic illustration of the octopus technique. (A) Preoperative marking showing the transverse abdominoplasty incision and the planned midline and lateral short vertical relaxing incisions. (B) Flap elevation with selective opening of vertical relaxing incisions to permit controlled tension redistribution. (C) Multidirectional release through several short vertical incisions allowing improved flap redraping and balanced closure. (D) Final appearance after layered closure and umbilical transposition, demonstrating tension-free wound approximation and low, symmetric scarring.

Figure 1 illustrates the preoperative marking design and incision pattern. Figure 2A shows the preoperative appearance, Figure 2B the postoperative outcome, and Figure 2C the intraoperative application of the Octopus Technique. Figure 3 presents representative preoperative and postoperative outcomes showing smooth abdominal contouring, symmetric scar placement, and effective tension-free healing. Figure 4 provides a stepwise schematic illustration of the Octopus Technique, including preoperative marking (Figure 4A), flap elevation with selective release (Figure 4B), multidirectional tension redistribution (Figure 4C), and final layered closure with umbilical transposition (Figure 4D). Written informed consent was obtained from all patients to participate in the study and to use their clinical images for publication.

## Results

A total of 12 consecutive female patients underwent abdominoplasty using the Octopus Technique. Baseline patient demographics and clinical characteristics are summarized in Table 1. The mean age was 40.3 years (range 33–50 years), and the mean BMI was 28.1 ± 1.2 kg/m^2^ (range 26.0–29.8 kg/m^2^). None of the patients were active smokers. Seven patients (58.3%) had a history of prior abdominal surgery, most commonly cesarean section. Five patients (41.7%) had at least one comorbidity, including hypertension (*n* = 3), diabetes mellitus type 2 (*n* = 2), and hypothyroidism (*n* = 2). Six patients were classified as ASA I and six as ASA II.

**Table 1.** Baseline patient demographics, clinical characteristics, and outcomes.

Pt	Age	Sex	BMI	Smoking	Prior Surgery	Comorbidities	ASA	Complications	FU (mo)
1	34	F	28.2	No	Cesarean section	None	I	None	12
2	41	F	29.5	No	Cesarean section x2	Hypothyroidism	II	Seroma (Grade I)	9
3	38	F	29.8	No	None	None	I	None	8
4	45	F	29.1	No	Cholecystectomy	HTN	II	None	6
5	36	F	27.4	No	Cesarean section	None	I	None	7
6	50	F	28.2	No	Hysterectomy	DM type 2, HTN	II	Superficial edge separation (Grade I)	6
7	33	F	26.9	No	None	None	I	None	5
8	42	F	27	No	Cesarean section x3	Hypothyroidism	II	Seroma (Grade I)	6
9	39	F	28.7	No	Appendectomy	None	I	None	4
10	47	F	29.5	No	Cesarean section x2	HTN	II	None	5
11	35	F	27.1	No	None	None	I	None	3
12	44	F	26	No	Cesarean section	DM type 2	II	None	7
**Mean**	**40.3**	**F**	**28.1**	**0/12**	**—**	**—**	**—**	**3/12 (25%)**	**6.5**

Pt = patient; BMI = body mass index; ASA = American Society of Anesthesiologists; FU = follow-up; mo = months; HTN = hypertension; DM = diabetes mellitus.

The mean follow-up duration was 6.5 months (range 3–12 months). Three relaxing incisions were used in 10 patients (83.3%), and five relaxing incisions were performed in 2 patients (16.7%) who had greater skin redundancy consistent with post-massive weight loss.

No major complications (Clavien-Dindo Grade III–V) were observed. Minor complications (Grade I) occurred in 3 of 12 patients (25%). These included two seromas that resolved with conservative management (observation without aspiration) and one superficial wound edge separation at the lateral aspect of the incision that healed with local wound care within 3 weeks. No cases of wound dehiscence, flap necrosis, hematoma, surgical site infection, or venous thromboembolism were recorded. All vertical relaxing incision sites (1–2 cm) healed well with silicone-based scar therapy, and none of the 12 patients developed hypertrophic or widened scars at these sites. While formal validated patient-reported outcome measures were not systematically collected, all patients expressed subjective satisfaction with their aesthetic outcomes at their last follow-up visit.

## Discussion

The Octopus Technique introduces a technical modification in abdominoplasty aimed at achieving a true tension-free closure. Excessive wound tension is a major contributor to complications such as dehiscence, flap necrosis, hypertrophic scarring, and suboptimal aesthetic outcomes [4]. By adding short vertical relaxing incisions to the traditional transverse incision, this method enables multidirectional tension redistribution and a more controlled, stepwise assessment of flap closure.

The incision pattern, inspired by the arms of an octopus, offers several practical advantages over conventional abdominoplasty. Traditional single-transverse closures rely solely on skin excision to relieve tension, which can create focal stress at the midline and lateral ends. In contrast, the Octopus Technique allows fine-tuned, vector-specific adjustment of closure forces through small, targeted incisions. This approach promotes even flap redraping, reduces scar widening, and enhances upper abdominal contour. Extending dissection laterally to the lower costal margins further improves flap mobility and tension balance compared with central dissection alone.

Vertical relaxing incisions also permit intraoperative evaluation of flap elasticity at multiple points, helping the surgeon determine where additional release is required. This reduces the likelihood of localized ischemia and optimizes wound perfusion. The technique can be particularly advantageous in extended abdominoplasty or combined contouring procedures, such as liposuction or belt lipectomy, where achieving symmetrical and tension-free closure can be challenging.

When compared with published complication rates for conventional abdominoplasty, the outcomes of the Octopus Technique in this preliminary series are encouraging. Published seroma rates for standard abdominoplasty range from 5–20%, wound dehiscence rates from 1–5%, and flap necrosis rates from 1–4% [2,8]. In our series of 12 patients, the seroma rate was 16.7% (2/12), wound dehiscence was 0%, and flap necrosis was 0%. While these results compare favorably, the small sample size precludes definitive statistical conclusions. Progressive tension sutures have been shown to reduce seroma rates to approximately 3–5% [3,5], and lipoabdominoplasty preserves perforator vascularity with complication rates comparable to standard techniques [7,9]. The Octopus Technique addresses a complementary dimension—namely, localized tension redistribution—and may be used in conjunction with these established methods.

While the Octopus Technique demonstrates promising potential, several limitations and risks must be acknowledged. The introduction of additional vertical incisions may theoretically increase the risk of visible secondary scars, although these are short, placed within the lower abdominal aesthetic unit, and typically heal inconspicuously when closed with fine intradermal sutures. Standardized undressed photographic documentation was not available for all patients in this preliminary series, which we acknowledge as a limitation; future studies will incorporate publication-grade standardized imaging. Operative time may be slightly longer due to additional marking and release steps; however, this is offset by simplified closure and potentially fewer wound-healing complications. Aesthetic concerns related to additional scars should be discussed with patients during preoperative planning, and meticulous closure is critical to minimize their visibility. Vertical scars were short (1–2 cm) and healed well with silicone-based scar therapy. None of the 12 patients developed hypertrophic or widened scars. Patients with keloid tendencies were counseled individually.

Compared with other established tension-reduction techniques—such as progressive tension sutures [3,5], which distribute forces effectively but prolong operative time; mesh reinforcement [6], which requires foreign material; and lipoabdominoplasty [7], which preserves vascularity but restricts flap mobility—the Octopus Technique offers a simpler, suture-free alternative that redistributes tension mechanically rather than through internal fixation or limited undermining.

The Octopus Technique may also be combined with other established methods to maximize tension control. For instance, using progressive tension sutures [3,5] alongside the vertical relaxing incisions could enhance internal flap adherence while maintaining multidirectional stress relief. Similarly, incorporating selective undermining as described in lipoabdominoplasty [7] may further preserve perforators and minimize dead space, offering both mechanical and vascular advantages. Such integration could synergistically optimize flap stability, reduce seroma formation, and promote more predictable wound healing.

## Limitations and trade-offs

Several limitations and trade-offs of the Octopus Technique warrant discussion. First, the additional operative time required for marking and performing the relaxing incisions is estimated at 10–15 min; however, this is offset by the simplified final closure and potentially reduced need for revision procedures. Second, based on our experience, approximately 3–5 cases are needed for the surgeon to become comfortable with intraoperative tension assessment and determining the optimal extent of relaxing incisions, representing a modest learning curve. Third, the vertical relaxing incisions (1–2 cm) represent additional scars that must be discussed with patients during preoperative counseling, although they are short, placed within the lower abdominal aesthetic unit, and typically heal inconspicuously. Fourth, the technique may not provide additional benefit in patients with minimal skin redundancy or low baseline closure tension, and patient selection remains important.

This study has inherent limitations as a retrospective, uncontrolled case series of 12 patients. The absence of a formal control group precludes direct comparison with conventional abdominoplasty. The mean follow-up of 6.5 months is insufficient to fully evaluate long-term scar maturation, contour stability, and recurrence of skin laxity. Furthermore, validated patient-reported outcome measures (PROMs) such as the BODY-Q were not prospectively collected, limiting the assessment of patient satisfaction and quality of life. While informal patient satisfaction was high, the absence of standardized assessment tools is a recognized shortcoming.

In our preliminary experience with 12 consecutive patients, the technique proved reproducible and safe, with no cases of wound dehiscence or flap necrosis. Minor postoperative events were limited to two small seromas managed conservatively and one superficial edge separation that healed with local dressings. While these early findings are encouraging, prospective comparative studies are warranted to evaluate complication rates, operative times, and patient satisfaction relative to established methods.

Future research should include prospective and comparative studies assessing both clinical and aesthetic outcomes of the Octopus Technique. A prospective, controlled comparative study between the Octopus Technique and conventional abdominoplasty, with matched cohorts and standardized outcome assessment, is planned. Key parameters for evaluation include wound healing quality (rates of dehiscence, seroma, and flap necrosis), scar characteristics (length, width, pigmentation, and patient-reported satisfaction), and operative time compared with traditional abdominoplasty. Quantitative measures such as tension distribution mapping, flap perfusion assessment using indocyanine green angiography, and pain or recovery scores would provide objective data on safety and efficacy. Future studies will incorporate a minimum 12-month follow-up with serial standardized photography, Patient and Observer Scar Assessment Scale (POSAS) scores, and assessment of contour symmetry at 3, 6, and 12 months postoperatively. Validated PROMs, including the BODY-Q (satisfaction with abdomen, scar, and overall outcome domains) and visual analog scales for aesthetic assessment, will be systematically applied. Collectively, these parameters would help define evidence-based refinements and standardize the use of the Octopus Technique in future surgical practice.

## Conclusion

The Octopus Technique introduces a simple and reproducible technical modification for achieving tension-free closure in abdominoplasty through short vertical relaxing incisions that allow multidirectional tension redistribution. By providing controlled, site-specific release without additional sutures or foreign material, it offers a practical alternative for improving wound healing and contour outcomes. In a preliminary series of 12 patients, the technique was safe and feasible, with no major complications observed over a mean follow-up of 6.5 months. Further prospective comparative studies with validated outcome measures are needed to validate its long-term benefits and determine its role alongside existing abdominoplasty techniques.
